# Resting-State Multi-Spectrum Functional Connectivity Networks for Identification of MCI Patients

**DOI:** 10.1371/journal.pone.0037828

**Published:** 2012-05-30

**Authors:** Chong-Yaw Wee, Pew-Thian Yap, Kevin Denny, Jeffrey N. Browndyke, Guy G. Potter, Kathleen A. Welsh-Bohmer, Lihong Wang, Dinggang Shen

**Affiliations:** 1 Image Display, Enhancement, and Analysis (IDEA) Laboratory, Biomedical Research Imaging Center (BRIC) and Department of Radiology, University of North Carolina at Chapel Hill, Chapel Hill, North Carolina, United States of America; 2 Brain Imaging and Analysis Center (BIAC), Duke University Medical Center, Durham, North Carolina, United States of America; 3 Joseph and Kathleen Bryan Alzheimer's Disease Research Center, Duke University Medical Center, Durham, North Carolina, United States of America; 4 Department of Psychiatry and Behavioral Sciences, Duke University Medical Center, Durham, North Carolina, United States of America; 5 Duke Institute for Brain Sciences, Duke University, Durham, North Carolina, United States of America; 6 Department of Medicine, Division of Neurology, Duke University Medical Center, Durham, North Carolina, United States of America; Beijing Normal University, China

## Abstract

In this paper, a high-dimensional pattern classification framework, based on functional associations between brain regions during resting-state, is proposed to accurately identify MCI individuals from subjects who experience normal aging. The proposed technique employs multi-spectrum networks to characterize the complex yet subtle blood oxygenation level dependent (BOLD) signal changes caused by pathological attacks. The utilization of multi-spectrum networks in identifying MCI individuals is motivated by the inherent frequency-specific properties of BOLD spectrum. It is believed that frequency specific information extracted from different spectra may delineate the complex yet subtle variations of BOLD signals more effectively. In the proposed technique, regional mean time series of each region-of-interest (ROI) is band-pass filtered (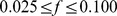 Hz) before it is decomposed into five frequency sub-bands. Five connectivity networks are constructed, one from each frequency sub-band. Clustering coefficient of each ROI in relation to the other ROIs are extracted as features for classification. Classification accuracy was evaluated via leave-one-out cross-validation to ensure generalization of performance. The classification accuracy obtained by this approach is 86.5%, which is an increase of at least 18.9% from the conventional full-spectrum methods. A cross-validation estimation of the generalization performance shows an area of 0.863 under the receiver operating characteristic (ROC) curve, indicating good diagnostic power. It was also found that, based on the selected features, portions of the prefrontal cortex, orbitofrontal cortex, temporal lobe, and parietal lobe regions provided the most discriminant information for classification, in line with results reported in previous studies. Analysis on individual frequency sub-bands demonstrated that different sub-bands contribute differently to classification, providing extra evidence regarding frequency-specific distribution of BOLD signals. Our MCI classification framework, which allows accurate early detection of functional brain abnormalities, makes an important positive contribution to the treatment management of potential AD patients.

## Introduction

Alzheimer's disease (AD) is one of the most prevalent dementia in older adults characterized by cognitive and intellectual deficits, which is serious enough to interfere daily life, without an effective treatment. It gets worse over time by gradually destroying brain cells, causing loss in memory and ability to reason, make judgements and communicate, and eventually causing death. AD is the most common type of dementia which accounts for 50 to 80 percent of dementia cases and definitive diagnosis can only be made with histopathological confirmation of amyloid plaques and neurofibrillary tangles. It has been reported that the incidence of AD doubles every five years after age of 65 [1] and 1 in every 85 persons will be affected by the disease by year 2050 [2]. The condition becomes even worse as life expectancy increases. With the aging of the world-wide population, this disease has become a serious problem and a huge burden to the healthcare system, especially in developed countries. Recognizing the urgent need to slow down or completely prevent the occurrence of a healthcare crisis worldwide, effort has been under way to administer and to develop effective pharmacological and behavioral interventions for delaying the onset and progression of the disease.

A significant body of literature [3]–[5] suggests that the pathological manifestation of AD begins many years before it can be diagnosed using cognitive tests. At the stage where symptoms can be observed, significant neurodegeneration of the human brain has already occurred. This provides an alternative approach to identify patients from normal aging based on neuroimaging data. Studies suggest that individuals with mild cognitive impairment (MCI), a prodrome of AD, tend to progress to probable AD at a rate of approximately 10% to 15% per year [6]–[8], compared with normal controls who develop dementia at a rate of 1% to 2% per year [9]. Thus, earlier diagnosis or prediction of MCI is important to possibly delay the onset of disease progression. However, compared to AD, MCI is more difficult to diagnose due to the subtlety of the involved cognitive impairment, especially in high functioning individuals who are able to maintain a positive public or professional profile without showing obvious cognitive impairment. It is hence crucial to develop algorithms that can identify subtle diagnostic biomarkers for early detection of MCI, so that early treatment can be administered to possibly delay the transition from MCI to AD or other dementias.

Functional connectivity is defined as the temporal correlation of a neurophysiological index measured in different brain areas [10], [11]. The neurophysiological index used in resting-state functional magnetic resonance imaging (fMRI) is the blood oxygenation level dependent (BOLD) signal. This signal exhibits low-frequency spontaneous fluctuations in the resting brain and shows a high degree of temporal correlation across widely separated brain regions. Resting-state fMRI yields new insights on how structurally segregated and functionally specialized brain regions are interconnected. Since the initial work by Biswal *et al.* [12], resting-state fMRI has been widely applied either in healthy subjects to study brain functional activities [13] or the pathological changes related to diseases such as multiple sclerosis [14], epilepsy [15], Schizophrenia [16], [17], depression [18]–[20], attention-deficit/hyperactivity disorder [21], MCI [22]–[24] and AD [25], [26]. It also been employed for graph-theory based parcellation of subunits in the brain [27]. One apparent clinical advantage of using resting-state fMRI rather than task-activation fMRI to investigate the influence of disease and/or medication on the brain is that no complicated experimental design is required. Experiments can be performed easily by patients who may have difficulties performing specific task inside the scanner, especially those with disorders exhibiting prominent cognitive degeneration, such as AD [11], [13].

Models of whole-brain connectivity, which comprise networks of brain regions connected either by anatomical tracts or functional associations, have drawn a great deal of interest recently due to the increasing reliability of network characterization through neurobiological meaningful and computationally efficient measures [28]–[30]. Network-based analyses on the constructed brain networks allow us to not only visualize the overall connectivity patterns among all the elements of the brain but also to quantitatively characterize responses of brain to external stimuli or pathological attacks [31]. For example, it has been demonstrated that both the structural and functional brain networks are organized in highly modular small-world architectures which allow transferring of information at a low wiring cost and high efficiency [29], [32]–[34].

One crucial step in resting-state fMRI signal analysis is temporal band-pass filtering. The purpose of this procedure is to minimize the effects of low frequency drift and high frequency noise. The frequency interval of band-pass filtering varies and depends on the application, but is normally within the interval of [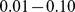 Hz]. The analysis of resting-state fMRI signals is normally performed on full-spectrum of the filtered signals, which might not be sensitive enough to delineate complex yet subtle pathological patterns related to the neurological disease. Such full-spectrum analysis on BOLD signal might cause subtle temporal changes to be averaged out, and thus deteriorate classification performance. A relatively more sensitive analysis, which is more effective in extracting subtly BOLD signal changes, is hence required.

The main goal of this study is to propose a novel neuroimaging-based classification framework for identifying MCI individuals from subjects undergoing normal aging using resting-state fMRI data. The proposed multivariate high-dimensional classification framework is developed based on the latest developments in graph theoretic analysis. In the proposed framework, a frequency-specific approach was employed to better characterize the subtle BOLD signal variations related to MCI and a graph measure was employed to extract topological information of brain functional connectivity networks. The utilization of frequency-sensitive approach is motivated by the inherent frequency specific property of low-frequency oscillations (LFO) that contributes differently to functional connectivity [35]. This can be accomplished by decomposing the BOLD spectrum into several frequency bands, to account for the subtle spatio-temporal changes of brain activity. Frequency specific research within the LFO range is originated by Buzsáki and colleagues who observed that neuronal oscillations are distributed linearly on the natural logarithmic scale [36], [37]. They suggested that independent frequency bands are generated by distinct oscillators, and each of them has specific properties and physiological functions. Recently, this concept has been extended to the fMRI studies [35], [38]–[40] by decomposing the fMRI signals with frequencies 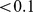 Hz into multiple bands [35], [39]–[41]. Zuo *et al.* demonstrated that the observed frequency-specific spatial structures are stable between repeat scans [40], while Baria *et al.* demonstrated similar frequency-specific spatial profiles over 195 subjects [39]. Accordingly, we hypothesize that by considering frequency bands individually, spatio-temporal profiles can be better characterized, particularly for complex yet subtle pathological patterns related to diseases, using the BOLD signal with multiple frequency bands. In our framework, the resting-state connectivity is characterized by multiple networks, each dominated by a BOLD oscillation with a specific frequency range for greater sensitivity in analyzing diseases such as AD.

Functional connectivity networks are constructed from each frequency sub-bands and their topological properties can be employed to describe the pathological patterns. In this study, clustering coefficients, which measure network topological properties, were extracted and fed into a feature selection mechanism to select the most discriminative subset of features before they were utilized to train a support vector machine (SVM) based classifier. Classification accuracy in this study was evaluated via leave-one-out cross-validation to ensure performance generalization. The classification accuracy obtained by the proposed method is 86.5%, which is an increase of at least 18.9% from the conventional methods which used a single frequency band. Specifically, we note that the area under receiver operating characteristic (ROC) curve is 0.863, indicating good diagnostic power of the proposed framework.

## Results

Performance of the proposed classification framework, which utilizes the frequency-specific multi-spectrum characterization, was compared with the full-spectrum characterization of the BOLD signal. Regions that were selected to effectively distinguish between MCI individuals and normal controls using the proposed framework are graphically displayed. The discriminative power of individual frequency sub-bands was also analyzed in detailed.

### Comparison Between Full- and Multi-Spectrum Schemes

Classification performance for the proposed framework was compared with the full-spectrum network characterization. The leave-one-out cross-validation training and testing procedures described in the Section **Method and Materials** were applied to both methods during comparison. The classification performance is summarized in Table 1.

**Table 1:** Classification accuracies and AUC values for full- and multi-spectrum network characterization methods using GM-masked and unmasked fMRI time series.

Approach	Accuracy	AUC
	(%)	
Unmasked+Full-Spectrum	56.76	0.5317
GM-Masked+Full-Spectrum	59.46	0.5433
Unmasked+Multi-Spectrum	67.57	0.6200
GM-Masked+Multi-Spectrum	86.49	0.8633

**Figure 1:**
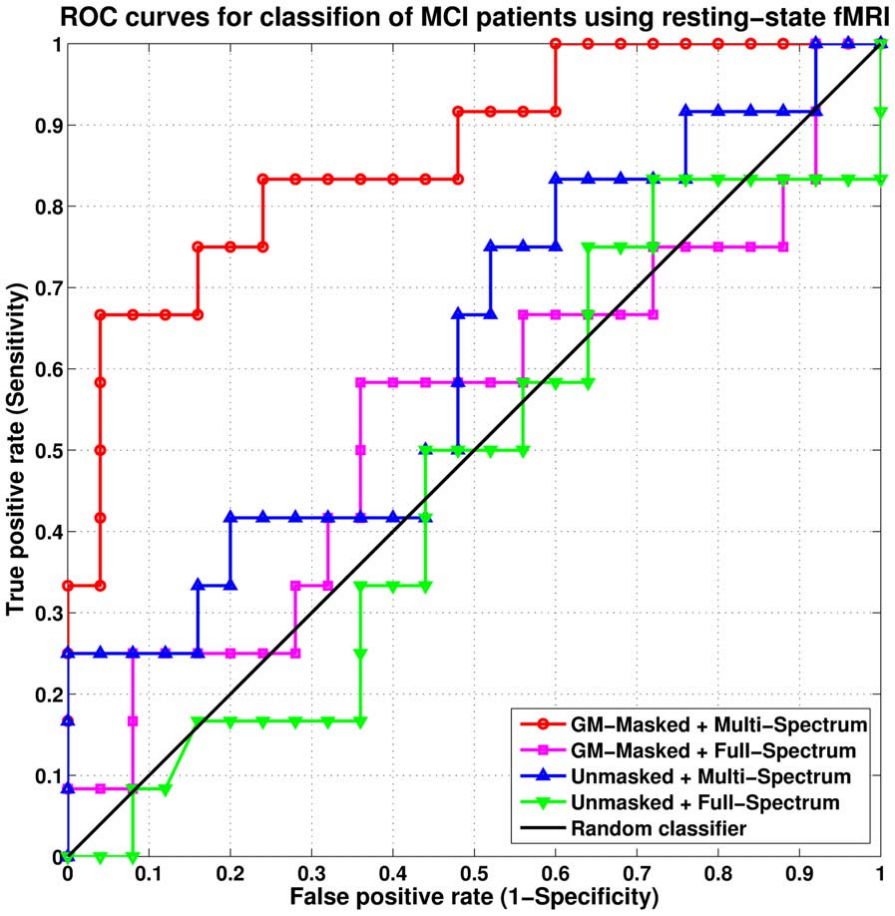
ROC curves for classification of MCI individuals using the resting-state fMRI.

In agreement with our hypothesis, the proposed multi-spectrum scheme provides pathologically more sensitive features by yielding significant improvement over the conventional full-spectrum scheme both in terms of classification accuracy and area under ROC curve (AUC), particularly after factoring out the effects of WM and CSF signals in the GM-masked fMRI time series. The classification accuracy increased by more than 18.9% while the AUC value increased by more than 0.24, indicating significant improvement in diagnostic power. The ROC curves of all compared conditions are shown in Figure 1.

### Discriminant Regions

The SVM recursive feature elimination (SVM-RFE) algorithm is utilized in the proposed framework to minimize the classification error in a backward sequential fashion by removing one feature at a time. The end result is a subset of most discriminant features which yields the best classification performance based on the training set. Since the proposed approach is evaluated in a nested leave-one-out fashion, the selected subset of features might be different for each leave-one-out fold. We hence define the most significant ROIs as the regions that are most frequently selected during the construction of optimal SVM models in the training stage. Specifically, for each training subset (which contains 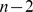 subjects) we first counted the frequency of ROIs selected in different 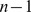 inner leave-one-out folds and then summed them up across all 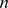 outer leave-one-out folds to obtain the final selection frequency. We finally ranked the ROIs according to their final selected frequency and ROIs with the highest frequency were considered as the most significant regions.

The most discriminant regions that were selected for classification were mainly located in prefrontal cortex areas and temporal lobes. The selected regions involved parts of frontal lobe such as rectus gyrus, orbitofrontal cortex and frontal gyrus, parts of temporal lobe such as temporal poles, amygdala and parahippocampal gyrus, superior occipital gyrus of occipital lobe and precuneus of parietal lobe.

The features selected by SVM-RFE in all leave-one-out cases and used for SVM model construction are listed in the Table 2.

**Table 2:** The selected most discriminant features.

Band	Most Discriminant Feature	Selected Frequency
Band2	Superior occipital gyrus right	1
Band2	Precuneus left	1
Band3	Orbitofrontal cortex (superior) left	4
Band3	Inferior frontal gyrus (opercular) right	19
Band3	Orbitofrontal cortex (medial) left	1230
Band3	Rectus gyrus left	55
Band3	Anterior cingulate gyrus left	8
Band3	Posterior cingulate gyrus left	2
Band3	Amygdala right	20
Band3	Temporal pole (superior) left	1
Band3	Temporal pole (superior) right	37
Band3	Temporal pole (medial) left	53
Band3	Right lobule VIIB of Cerebellar hemisphere	2
Band4	Rectus gyrus left	15
Band4	ParaHippocampal gyrus left	64

(Band1 = [0.025–0.039 Hz], Band2 = [0.039–0.054 Hz], Band3 = [0.054–0.068 Hz], Band4 = [0.068–0.082 Hz], Band5 = [0.082–0.10 Hz]).

**Figure 2:**
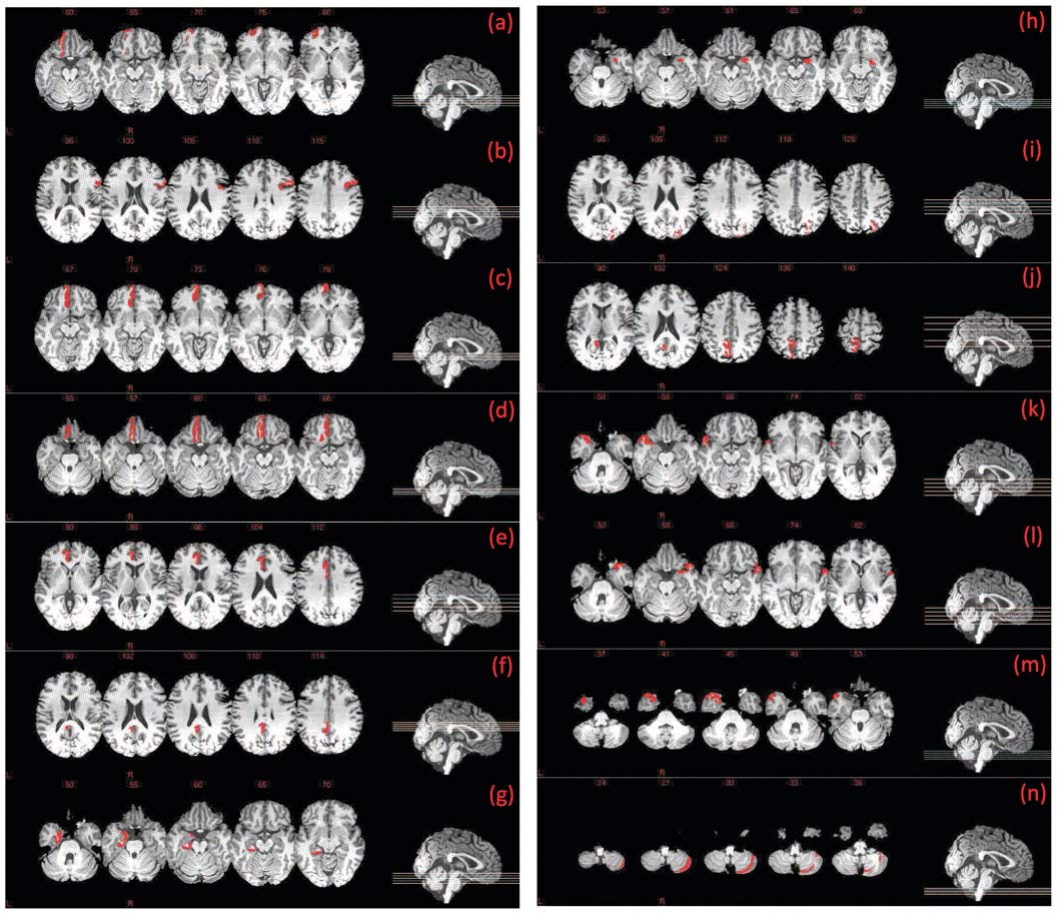
The most discriminant ROIs selected for classification. ((a) Orbitofrontal cortex (superior) left, (b) Inferior frontal gyrus (opercular) right, (c) Orbitofrontal cortex (medial) left, (d) Rectus gyrus left, (e) Anterior cingulate gyrus left, (f) Posterior cingulate gyrus left, (g) Parahippocampal gyrus left, (h) Amygdala right, (i) Superior occipital gyrus right, (j) Precuneus left, (k) Temporal pole (superior) left, (l) Temporal pole (superior) right, (m) Temporal pole (middle) left, and (n) Right lobule VIIB of cerebellar hemisphere).

The most discriminant ROIs obtained using the proposed classification framework are graphically displayed in Figure 2.

### Discriminative Power of Individual Frequency Sub-Bands

In order to investigate which frequency ranges were most predictive, we performed MCI classification using features derived from each individual frequency sub-band. The same leave-one-out cross-validation classification procedure was applied for each individual frequency sub-band and their performance is summarized in Table 3.

**Table 3:** Comparison of classification performance for multi-spectrum and individual frequency sub-bands.

Approach	Accuracy	AUC
	(%)	
Band1	64.87	0.6367
Band2	67.57	0.6781
Band3	83.78	0.8267
Band4	70.27	0.7067
Band5	64.87	0.6513
Multi-Spectrum	86.49	86.33

(Band1 = [0.025–0.039 Hz], Band2 = [0.039–0.054 Hz], Band3 = [0.054–0.068 Hz], Band4 = [0.068–0.082 Hz], Band5 = [0.082–0.10 Hz]).

It is clearly observed that each individual frequency sub-band performed differently in classifying MCI, indicating their different discriminative power. Frequency Band3 ([0.054–0.068 Hz]) performed the best among all frequency sub-bands, though still slightly inferior than the proposed multi-spectrum method, both in terms of classification accuracy and AUC values. The other frequency sub-bands performed significantly worse than the Band3, in the order of Band4 ([0.068–0.082 Hz]), Band2 ([0.039–0.054 Hz]), Band5 ([0.082–0.100 Hz]) and Band1 ([0.025–0.039 Hz]). Band5 and Band1 performed the worst and no obvious differences between them. Band4 and Band2 performed slightly better than Band5 and Band1. This result explains why only features from Band2, Band4, and predominantly Band3 were selected during feature selection step.

## Discussion

This paper investigated the diagnostic power of functional connectivity networks, derived from resting-state fMRI, for the identification of individuals with MCI from normal aging subjects. The proposed high-dimensional pattern classification framework employed an efficient frequency-specific multi-spectrum characterization of resting-state fMRI time series for accurate identification of MCI individuals. Classification accuracy was evaluated via leave-one-out cross-validation to ensure performance generalization. The classification accuracy obtained by the proposed method, involving frequency-specific multi-spectrum characterization and graph theoretic analysis, was 86.5%, which was significantly higher than the conventional full-spectrum approach. The AUC value of the proposed method was 0.863, indicating good diagnostic power, especially in view of the relatively limited number of samples available in this study. Conventional approach, which is either less sensitive to BOLD signal changes or vulnerable to noise effects, can only provide low to moderate performance as indicated by their relatively smaller AUC values. It was also found that the classification performance of individual frequency sub-bands is different indicating frequency-specific spatio-temporal information distribution in BOLD signals.

A strength of our assessed cohort is that it allows us to study MCI at a relatively mild stage of cognitive impairment as indicated by very minor differences in MMSE scores between MCI patients and normal aging subjects. This provides an important distinction between our study and other studies that involve patients reporting to the clinic with significant memory and cognitive complaints.

Multi-spectrum characterization provides a frequency-specific description regarding the variability of the BOLD signal by decomposing the band-pass filtered time series into several smaller frequency intervals. This description reveals subtle variations of the BOLD signal and thus provides more sensitive characterization of neuronal activities during resting condition, particularly for comparison between baseline and affected BOLD signals. This is justified by the improvement of classification accuracy yielded by the multi-spectrum characterization when compared with the conventional full-spectrum approach. Further decomposing of the band-pass filtered time series, however, is restricted by the acquisition sampling frequency. It was found that each individual frequency sub-band contributed differently to classification, with Band3 ([0.054–0.068 Hz]) demonstrating the highest discriminative power. Our results based on multi-spectrum characterization are supported by findings in studies that examine the frequency-specific distribution of BLOD signal fluctuations. Many studies that examined the resting-state fMRI data by decomposing the the BOLD frequency bandwidth into separate length bands [35], [39], [40], [42] indicate that resting-state BOLD oscillations exhibit frequency-specific anatomical constrained spatial structure in the human brain [39]. These frequency-specific structure was proven to be stable between repeat scans [40] and similar finding was repeated using large dataset [39]. Baria *et al.* [39] also demonstrated that power for different frequency bands varies by anatomical and functional properties of the brain. Our proposed classification framework combines different yet complementary spatio-temporal information conveyed by each individual frequency sub-band and thus enhances the classification performance of MCI. However, further analysis is required to determine the frequency band that is most effective for classification.

The brain regions that are selected for accurate detection of individuals with MCI includes portions of the prefrontal cortex, orbitofrontal cortex, temporal lobe, and parietal lobe, which have already been extensively reported in previous studies. These included: parts of prefrontal regions [43], the inferior frontal gyrus and cingulate areas [44], parts of parietal and posterior cingulate regions [45], the medial temporal lobe areas and posterior cingulate that are core regions of default mode network [3], [46], [47], the medial temporal lobe and parahippocampal regions [48], posterior cingulate cortex and middle frontal cortex [49], the medial temporal lobe [50]–[52]. It is worth noting that our proposed method is blind to the prior knowledge of brain regions associated with MCI or AD. Yet, the selected regions coincide well with those reported in the literature.

Our findings of relative asymmetric pattern, i.e., more regions in the left hemisphere were selected in training stage, is in line with some previous studies using SPECT and PET data [53], [54]. Functional studies in [55] reported a left-sided hypometabolism in patients with AD while [56] reported a better discrimination between MCI individuals and healthy controls in the left-hemisphere. The findings may suggest that alteration of brain functions in left-hemisphere is occurred earlier in the disease process than the right-hemisphere. However, there is no strong evidence regarding this observation since non-pathological factors such as patient selection and post-processing procedure may cause for finding differences. This asymmetric pattern is interesting and merits further research.

It is noteworthy that the framework proposed herein is based on the data-driven concept, i.e., the set of brain measurements which optimally differentiates between MCI individuals and cognitively normal individuals cannot be known *a priori*, but can only be determined from the data. Furthermore, the leave-one-out cross-validation is used in this study to guard against data overfitting, a persistent problem in high dimensionality analyses of datasets with relatively small sample sizes.

One of the methodological issues regarding post-processing of resting-state fMRI analysis is the choice of temporal filtering algorithms. Wavelet [57] and Fourier-based approaches [34], [58] have been proposed for the characterization of BOLD signal fluctuations and a good agreement, with correlations larger than 0.9, between these two approaches has been found across subjects and parcellation scales [57].

Another methodological issue that may affect the classification performance is the global correction of BOLD signal based on whole-brain signal. The whole-brain signal is defined as the average time series over all voxels in the brain and is typically removed by regression from time series at each voxel, after which the residual time series is used for further analysis [59]. Whole-brain signal regression is associated with the artificial emergence of negative correlations. It is currently not known to what extent the whole-brain signal correlates with signals of true neurophysiological origin, or how activation of multiple coherent networks during rest may contribute to the whole-brain signal [60].

Our current study is limited by the small size of available samples. Given a small sample size, statistical power is of potential concern. Although the leave-one-out cross-validation accuracy obtained may be optimistic, the limited sample size does not allow us to explore other cross-validation techniques, since the nonlinear SVM classifier will then be under-trained. Our dataset is quite diverse, and it includes both sexes and all ages between 55 to 84 for MCI patients and 55 to 88 for normal controls. However, the results obtained have to be verified in the future with larger datasets to reduce individual effects and to ensure the effectiveness of the proposed technique.

In summary, resting-state fMRI has the potential for greatly increasing the clinical utility of the fMRI for diagnosis of disorders such as AD. A novel high-dimensional pattern classification method, which is based on BOLD signal contrast, has been proposed to identify individuals with MCI from normal controls. The proposed technique employs a frequency-specific multi-spectrum network characterization of the fMRI regional mean time series to effectively delineate the functional connectivity patterns at a whole brain level. Significant improvements and promising results indicate that the proposed classification framework can potentially serve as a complementary approach to clinical diagnosis of alteration in brain functions associated with cognitive impairment, especially at the early stages.

## Materials and Methods

### Participants and Data Acquisition

All the subjects used in this study were recruited by the Duke-UNC Brain Imaging and Analysis Center (BIAC), Durham, North Carolina, USA. Written consent was obtained from all participants, and the experimental protocols were approved by the institutional ethics board at Duke University Medical Center in compliance with the Health Insurance Portability and Accountability Act. This cohort involved of 37 participants, 12 MCI patients and 25 socio-demographically matched healthy controls. All the recruited subjects were diagnosed by expert consensus panels at the Joseph and Kathleen Bryan Alzheimer's Disease Research Center (Bryan ADRC) and the Department of Psychiatry at Duke University Medical Center. Diagnosis was made by consensus with the ultimate decision by a board-certified neurologist in concert with available data from a battery of general neurological examination, neuropsychological assessment evaluation, collateral and subject symptom and functional capacity reports. The neuropsychological battery the Bryan ADRC used was a revised Consortium to Establish a Registry in Alzheimer's Disease (CERAD) which included: 1) Mini-Mental State Examination (MMSE); 2) immediate and delayed verbal memory (Logical Memory subtest of the Wechsler Memory Scale-Revised); 3) visual immediate memory (Benton Visual Retention Test); 4) verbal initiation/lexical fluency (Controlled Oral Word Association Test from the Multilingual Aphasia Examination); 5) attentional/executive functions (Trail Making Test, Symbol Digit Modality Test, Digit Span sub-test of the Wechsler Adult Intelligence Scale-Revised, and a separate ascending Digit Span task modeled after the Digit Ordering Test); 6) premorbid verbal ability (Shipley Vocabulary Test); 7) Finger Oscillation Grooved Pegboard; and 8) Self Rating of Memory Function.

Conformation of diagnosis for MCI if subjects met the following inclusion criteria: 1) age 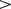
55 years and any race; 2) recent worsening of cognition, but still functioning independently; 3) MMSE score between 24 and 30; 4a.) score 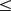
−1.5 SD on at least two Bryan ADRC cognitive battery memory tests for single-domain amnestic MCI; or 4b.) score 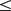
−1.5 SD on at least one of the formal memory tests and score 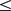
−1.5 SD on at least one other cognitive domain task (e.g., language, visuospatial-processing, or judgment/executive function) for multi-domain MCI; 5) 4 or lower for baseline Hachinski score; 6) does not meet the NINCDS-ADRDA or DSM-IV-TR criteria for dementia; 7) no psychological symptoms or history of depression; and 8) capacity to give informed consent and follow study procedures.

Similarly, all healthy controls met the following criteria: 1) age 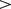
55 years and any race; 2) adequate visual and auditory acuity to properly complete neuropsychological testing; 3) no self-report of neurological or depressive illness; 4) shows no evidence of depression based on the Diagnostic Interview Schedule port based on the Diagnostic Interview Schedule portion of the Duke Depression Evaluation Schedule; 5) normal score on a non-focal neurological examination; 6) a score 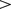
−1 SD on any formal memory tests and a score 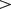
−1 SD on any formal executive function or other cognitive test; and 7) demonstrates a capacity to give informed consent and follow study procedures. In order for safety purposes and minimizing biases, subjects were excluded from the study if they have: 1) any of the traditional MRI contraindications, such as foreign metallic implants or pacemakers; 2) a past head injury or neurological disorder associated with MRI abnormalities, including dementia, brain tumors, epilepsy, Parkinson's disease, demyelinating diseases, *etc.*; 3) any physical or intellectual disability affecting completion of assessments; 4) documentation of other Axis I psychiatric disorders; and 5) any prescription medication (or nonprescription drugs) with known neurological effects. Noteworthy that the diagnosis of all cases were made on clinical grounds without reference to MRI.

An 3.0 T GE scanner (Signa EXCITE, GE Healthcare) was used in scanning process to acquire resting-state fMRI volumes. Resting-state functional images of each participant were acquired axially parallel to the horizontal plane connecting the anterior and posterior commissures (AC-PC line) with echo time (TE) = 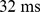, repetition time (TR) = 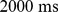 and flip angle = 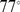. The acquisition matrix was (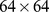) with a rectangular FOV of (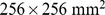), resulting in a voxel resolution of 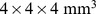. A total of 34 slices were acquired using a SENSE inverse-spiral pulse sequence in the same plane as the low resolution T1-weighted images. There were 150 volumes acquired per scan in all participants. All the subjects were told to keep their eyes open and stare at a fixation cross in the middle of the screen during scanning, which lasted for 5 minutes. As we know, neurons get excited to changing stimuli across time. But when the stimuli such as the little cross sign in this study was presented steadily without changing across the five minutes period, the neural excitation related to the stimuli can vanish quickly. Hence, this can ensure subjects not falling into sleep and avoid saccade-related activation which is unavoidable if eyes were closed. The same scanner was used to acquire the T1-weighted anatomical MRI images using the following parameters: TE = 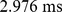, TR = 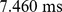 and flip angle = 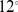. The acquisition matrix was (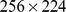) with a rectangular FOV of (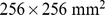), resulting in slice thickness of 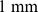. A total of 216 slices were acquired using the FSPGR ASSET sequence. Demographic information of the participants involved in this study are shown in Table 4.

**Table 4:** Demographic and clinical information of the participants involved in this study.

Group	MCI	Normal
No. of subjects	12	25
No. of males	6	9
Age (mean 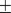 SD)	75.0 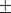 8.0	72.9 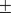 7.9
Years of education (mean 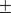 SD)	18.0 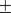 4.1	15.8 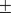 2.4
MMSE (mean 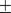 SD)	28.5 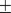 1.5*	29.3 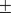 1.1

* One of the patients does not have a MMSE score.

### Method

**Overview of Methodology:** The key of our proposed multivariate high-dimensional classification framework involves an efficient characterization of resting-state fMRI time series via a **two-fold** description:

- **Frequency-specific multi-spectrum characterization** - to quantify relatively subtle changes of BOLD signal by decomposing the mean time series of each ROI into five distinct frequency sub-bands; and
- **Graph theoretic analysis** - to characterize topological properties and strengths of brain functional connectivity networks through neurobiologically meaningful and computationally efficient measures.

**Figure 3:**
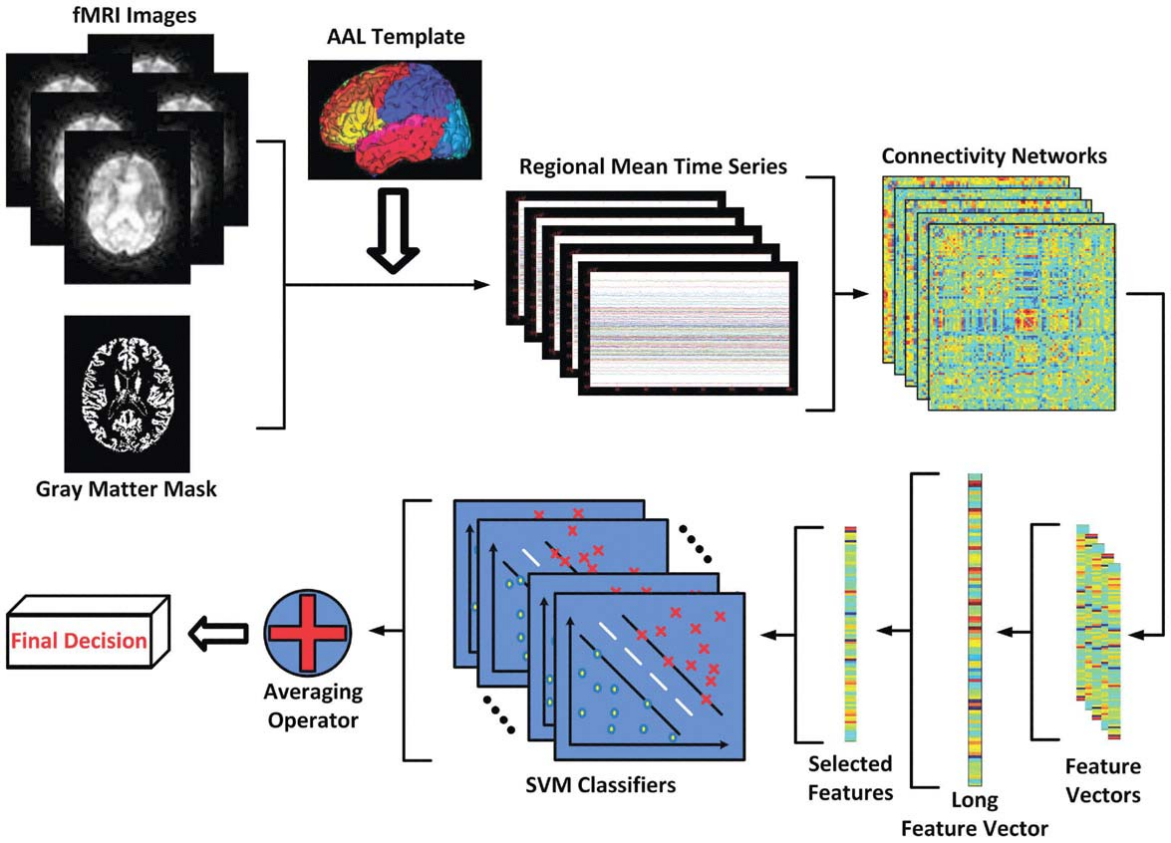
Schematic diagram of the proposed MCI classification framework, which employs a multi-spectrum characterization of the resting-state fMRI time series.

An overview of the proposed MCI classification framework is summarized graphically in Figure 3.

Post-processing of the fMRI images of the resting-state fMRI images was performed using the Statistical Parametric Mapping software package (SPM8, http://www.fil.ion.ucl.ac.uk.spm). Specifically, the first 10 acquired fMRI images of each subject were discarded to ensure magnetization equilibrium. Then, the remaining 140 images were corrected for the staggered order of slice acquisition that was used during echo-planar scanning. The correction makes the data on each slice correspond to the same point in time. The interpolated time point was chosen as the TR/2 time to minimize relative errors across each TR in the study. After acquisition time delay correction, we realigned the slice timing corrected fMRI time-series of each subject using a least squares approach and a 6 parameters (rigid body) spatial transformation [61]. The first image of each subject was used as the reference to which all subsequent scans were realigned. This realignment removed the head-motion artifacts in fMRI time-series. For all subjects used in this study, there were no significant group differences in head-motion. After realignment, the images were resliced such that they match the first image voxel-for-voxel.

Before constructing the functional connectivity networks, we minimized the effects or contributions from WM and CSF signals by generating GM-masked fMRI images. Specifically, we first performed tissue segmentation on the T1-weighted image of each subject to obtain segmentation images representing three different brain tissues, i.e., GM, WM and CSF. The fMRI images of each subject were then masked with their respective GM masks. After that, we parcellated the brain space into 116 ROIs by warping the Automated Anatomical Labeling (AAL) template [62] to these GM-masked fMRI images using deformation fields estimated from T1-weighted images. For each subject, the representative time series of each individual region was obtained by averaging the GM-masked fMRI time series over all voxels in that particular region. Temporal band-pass filtering of frequency interval (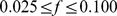 Hz) was then performed to minimize the effects of low-frequency drift and high-frequency noise. This frequency interval is further decomposed into five equally divided, non-overlapping frequency sub-bands.

For each frequency sub-band, we constructed a functional connectivity network by utilizing Pearson correlation to measure the interregional synchronization in BOLD signal. No global signal regression was performed in this study to avoid artifacts resulting from negative correlations. For each sub-band connectivity network, we computed the clustering coefficient of each node with respect to the other nodes in its subnetwork as feature for classification. For each subject, features from all connectivity networks were concatenated to form a large feature pool. Elements from this large feature pool were first ranked according to their Pearson correlation with respect to the clinical labels, and were further sieved to select the most discriminant features subset using a wrapper-based feature subset selection method, i.e., the SVM-RFE algorithm [63], [64]. Finally, nonlinear SVMs were trained using the selected subset of features. The training process was repeated for all subjects in the dataset in a leave-one-out fashion. Given an unseen testing sample, the final decision was determined by averaging the outcome from all built SVM classifiers.

**Frequency-Specific Multi-Spectrum Characterization:** For each subject, the mean time series of each individual ROI was obtained by averaging the GM-masked fMRI time series over all voxels in that particular ROI. Temporal band-pass filtering with frequency interval (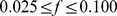 Hz) was then performed on the mean time series of each individual ROI. This frequency interval is commonly employed to characterize connectivity patterns of spontaneous, low-frequency fluctuations (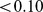 Hz) of the BOLD signal in resting-state fMRI analysis since the fMRI dynamics of neuronal activities are most salient within this frequency interval. It provides a reasonable trade-off between avoiding the physiological noise associated with higher frequency oscillations [65], the measurement error associated with estimating very low frequency correlations from limited time series [66], and the magnetic field drifts of the scanner [67].

In order to extract complex, yet subtle pathologies associated with MCI, we proposed to employ a frequency-specific multi-spectrum characterization of the regional mean time series, which utilizes multiple frequency sub-bands, in contrast to the conventional full-spectrum description, to construct functional connectivity networks. We hypothesis that by decomposing the BOLD spectrum into smaller frequency bands and performing the analysis using these sub-bands will provide a more sensitive characterization on spatio-temporal information of brain activity. In [40], based on Buzsáki's framework, Zuo *et al.* decomposed LFO into four frequency bands: slow-5 ([0.01–0.027 Hz]), slow-4 ([0.027–0.073 Hz]), slow-3 ([0.073–0.198 Hz]) and slow-2 ([0.198–0.25 Hz]), and they found that only slow-5 and slow-4 are associated with resting-state functional connectivity while slow-3 and slow-2 are associated with respiratory and cardiac signals. It was also shown that, compared with slow-5, slow-4 has higher test-retest reliability [40]. In another study, BOLD signal was decomposed into four frequency bands: LF ([0.01–0.05 Hz]), MF1 ([0.05–0.10 Hz]), MF2 ([0.10–0.15 Hz]) and HF ([0.15–0.20 Hz]), and it was found that the low frequencies (LF and MF1) contained larger average power spectral density than the high frequencies (MF2 and HF) [39]. In another study which decomposed BOLD signal with frequency (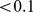 Hz) into four bands, it was found that small-world properties were salient in the frequency ranges [0.06–0.11 Hz], [0.03–0.06 Hz] and [0.01–0.03 Hz], with the most salient range [0.03–0.06 Hz] [38]. Accordingly, in the proposed framework, we incorporated the findings from these studies by restricting the analysis within the frequency range (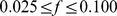 Hz). To better characterize the subtle changes of functional connectivity, we decomposed the band-pass filtered GM-masked mean time series of each region into five frequency bands using the Fast Fourier transform (FFT). The five distinct, equally divided frequency bands were: Band1 ([0.025–0.039 Hz]), Band2 ([0.039–0.054 Hz]), Band3 ([0.054–0.068 Hz]), Band4 ([0.068–0.082 Hz]), and Band5 ([0.082–0.100 Hz]).

**Estimation of Interregional Functional Connectivity:** Functional connectivity that examines interregional correlations in neuronal variability [10] was measured using a pairwise Pearson correlation coefficients between a given pair of ROIs. Given a set of 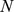 random variables, the Pearson correlation matrix is a symmetric matrix in which each off-diagonal element is the correlation coefficient between a pair of variables.

**Figure 4:**
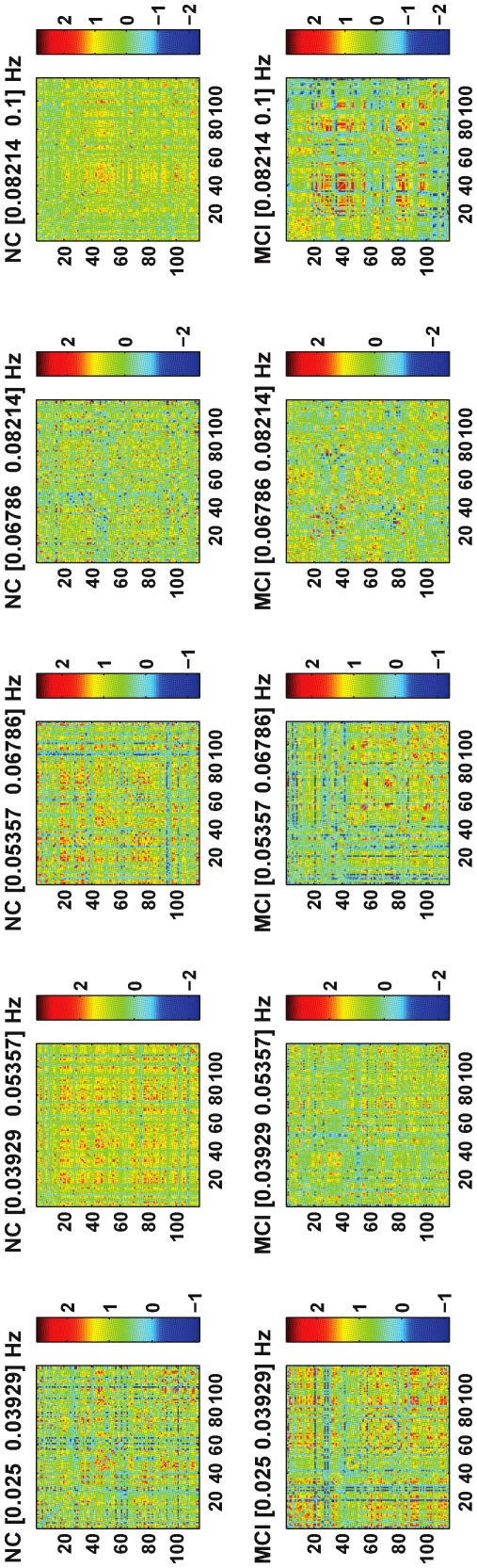
Multi-spectral functional connectivity maps for a normal control (NC) and an MCI individual.

We considered the brain regions as a set of nodes and the correlation coefficients as signed weights on the set of edges. A Fisher's 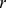-to-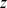 transformation was applied on the computed Pearson correlation matrix to improve the normality of Pearson correlation coefficients. Formulation of the transform is given as
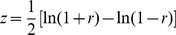
(1)where 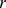 is the Pearson correlation coefficient and 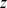 is normal with standard deviation 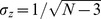. The functional connectivity networks are represented in the form of 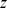-maps. Examples of the functional connectivity maps constructed using the proposed multi-spectrum characterization for one normal control (NC) and one MCI individual are shown in Figure 4.

### Feature Extraction, Feature Selection and Classification

In this study, we employed the weighted-graph local clustering coefficients [68], [69] - a segregation measure in network analysis that quantifies the degree to which nodes in a network tend to cluster together - to extract neuronal dynamics from the constructed functional connectivity networks. In each connectivity network, the weighted local clustering coefficient between each ROI and the remaining ROIs was computed, resulting in 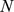 clustering coefficients for each 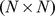 network. For each network, the weighted local clustering coefficients between each ROI and the remaining ROIs are computed as
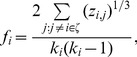
(2)where 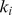 is the number of ROIs that are connected to the 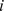-th ROI, 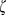 is the subnetwork comprising nodes directly connected to the 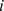-th ROI, and 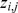 is the parameter value between the 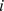-th ROI and 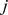-th ROI. It is noteworthy that we only considered the positive 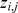 values during the computation of clustering coefficients. Since AAL template was used in the anatomical parcellation, 116 clustering coefficients were obtained from each network, and there was a total of 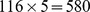 coefficients for each subject.

These clustering coefficients form a feature pool from which we employed a hybrid feature selection method that combined the benefits of maximum-relevance and wrapper-based feature selection methods to determine the most discriminant features subset [70]–[73]. Relevancy of a feature to classification was quantified using the Pearson correlation coefficients. From a subset of features with the highest Pearson correlation coefficient values, a set of most discriminant features were then selected using a well-known wrapper-based feature selection method, i.e., the SVM-RFE algorithm [63], [64]. This algorithm is a backward sequential selection method that remove one feature at a time. For removal of a particular feature, SVM-RFE ensures that the smallest classification error is achieved, compared to removing other features. Note that SVM-RFE is performed via a leave-one-out procedure to estimate the generalization error with respect to the number of features and to minimize this error in order to choose the optimal combination of features. These selected features are considered as the most discriminant features that provide the best classification performance.

Classification performance of the proposed framework was evaluated using a nested full leave-one-out cross-validation strategy to ensure a relatively unbiased estimate of the generalization power of the classifiers to new subjects. We adopted the SVM classifier with non-linear radial basic function (RBF) kernel in the proposed framework. In each leave-one-out case, one subject was first left out as the testing subject, and the remaining subjects were used for feature extraction, feature selection and classifier training. Then, second or inner leave-one-out was applied within the training set, to build an ensemble classifier whose parameters were automatically optimized via grid search. Specifically, for 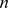 total number of subjects involved in the study, one was left out for testing, and the remaining 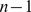 were used for training. From these 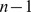 samples, 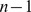 different training subsets were formed by each time leaving one more sample out, giving us 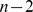 subjects in each training subset. For each training subset, feature extraction and feature selection were performed for each combination of SVM parameters, i.e., the penalty factor 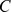 and the 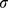 value of RBF kernel. The SVM parameters were varied across certain range during grid search. The performance of each combination of SVM parameters and the selected features was evaluated using the second left out subject. The best performed combination was used to construct the optimal SVM model for future classification. This procedure was repeated 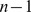 times, once for each training subset. This procedure allowed us to select parameters which maximize the area under receiver operating characteristic (ROC) curve. When the completely unseen (totally left out during the entire training and parameter optimization process) test sample was to be classified, all 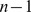 classifiers were used, and their outcomes were combined using an averaging operator to provide the final classification decision. This process was repeated 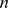 times, each time leaving out a different subject, finally leading to an overall cross-validation classification accuracy.

### Evaluation of The Proposed Framework

Performance of the proposed frequency-specific multi-spectrum scheme is compared with the conventional full-spectrum scheme. The proposed framework is evaluated under different settings, i.e., GM-masked+multi-spectrum (proposed), unmasked+multi-spectrum, GM-masked+full-spectrum, and unmasked+full-spectrum. This will provide quantitative evidences to test our hypothesis that multi-spectrum analysis is more sensitive than commonly used full-spectrum analysis in capturing subtle variation of BOLD signals that is induced by pathological attacks, as well as its contribution to the prediction accuracy.

The features are determined based on their contribution to the classification performance using cross-validation. The selected features will give an indication of the brain regions that potentially experience the greatest functional changes in BOLD signal. Further analysis on individual frequency sub-bands also been performed to explore the contribution of each individual sub-band in MCI classification as well as their relationships with the selected features.
